# circRNA, a novel diagnostic biomarker for coronary heart disease

**DOI:** 10.3389/fcvm.2023.1070616

**Published:** 2023-02-01

**Authors:** Xiao Tong, Xinyi Zhao, Xuan Dang, Yan Kou, Junjie Kou

**Affiliations:** Department of Cardiology, The 2nd Affiliated Hospital of Harbin Medical University, The Key Laboratory of Myocardial Ischemia, Chinese Ministry of Education, Harbin, China

**Keywords:** diagnostic marker, plasma exosome, coronary heart disease, ceRNA network, differentially expressed genes

## Abstract

**Objective:**

This study aimed to identify the potential diagnostic biomarkers of coronary heart disease (CHD) from exosome-derived circRNA.

**Methods:**

The microarray data of circRNA derived from the exosomes of patients with CHD and mRNA in acute myocardial infarction was retrieved from exoRBase website and GEO database (GSE61144), respectively, to identify the differentially expressed genes (DEGs). Our findings detected the differentially expressed circRNAs and mRNAs and predicted their correlation with microRNAs using the microRNA target prediction website, thus ascertaining the corresponding circ-microRNA and micro-mRNAs. Then, we performed systematic Gene Ontology (GO) and Kyoto Encyclopedia of Genes and Genomes (KEGG) analysis on the differentially expressed mRNA. Protein-Protein Interactions (PPI) of these DEGs were examined using STRING. The receiver operator characteristic (ROC) curve was used to validate the diagnostic efficacy of circRNA in patients with CHD. Finally, the RNAs identified in this study were verified by quantitative real-time polymerase chain reaction (qRT-PCR).

**Results:**

A total of 85 differentially expressed circRNAs (4 up-regulated and 81 down-regulated) were identified by screening the circRNAs in exosome of CHD patients. Based on the prediction data of circRNA, mRNA, and the corresponding microRNA, a ceRNA network was constructed, including 7 circRNA nodes, 5 microRNA nodes, and 2 mRNA nodes. Finally, validated by qRT-PCR testing, we found circRNA0001785, circRNA0000973, circRNA0001741, and circRNA0003922 to be the promising candidate for the effective prediction of CHD. These potential diagnostic markers can provide insight for further research on the occurrence of CHD or even acute coronary syndrome (ACS).

## 1. Introduction

Ischemic heart disease (including acute myocardial infarction) is the leading cause of death globally and accounted for 17.3% of all deaths in 2016 (1, 2). It is well known that myocardial injury leads to narrowing of coronary arteries and exacerbates oxygen deficiency in myocardial cells (3). Although PCI and coronary artery bypass grafting are widely used to treat patients with acute myocardial infarction, post-operative complications and reduced cardiac function remain inevitable (4). Elucidating the mechanism underlying atherosclerotic plaque formation and triggering of plaque rupture may facilitate the development of treatments for patients with CHD.

Exosomes are intracellular membrane-bound vesicles with diameters of 30–150 nm that originate from a variety of intracellular cell types and transfer their bioactive molecules between cells (5). Exosomal contents change in response to environmental stimuli on their parental cells (5–7), thus modulating downstream biological effects of the exosomes. Moreover, exosomes can deliver their cargo, such as functional nucleic acids (microRNAs, mRNAs, and other RNA types), to recipient cells, thereby regulating these cells at the post-transcriptional level (8–10). Therefore, exosomes are intercellular signaling factors that can deliver bioactive proteins, lipids, and RNA species in both paracrine and autocrine fashions (11, 12). For example, exosomes isolated from human atherosclerotic plaques were shown to directly transfer functional ICAM1 to target cells, and plaque exosomes from symptomatic patients could induce monocyte adhesion and migration more strongly than those from asymptomatic patients, indicating functional differences among these exosomes (13). Therefore, the detection of differentially expressed genes of exosomal origin enables a more accurate determination of diagnostic biological markers of disease.

Circular RNA (CircRNA) is a novel endogenous non-coding RNA, characterized by a covalent closed-loop structure called post-clipping through a special type of alternative splicing (14). In recent years, the development and application of microarray technology, RNA sequencing analysis (RNA-seq), and new bioinformatic methods have led to the discovery of many circRNAs (15–19). For example, has-circ-0124644 can be used as a diagnostic biomarker of CHD (20, 21), and has-circ-0005870 can be used as a diagnostic biomarker of hypertension (22). In addition, some circRNAs have also been identified as new prognostic biomarkers for patients with heart failure after cardiac infarction (23–25). Similarly, the enrichment and stability of circRNAs in exosomes were also identified in body fluids, such as human blood, saliva, and cerebrospinal fluid, indicating that these Exo-circRNAs have potential applications as disease biomarkers and novel therapeutic targets (20). Some databases also indicate that the number of circRNAs in exosomes is even higher than the number of mRNAs. However, the role of exosomal circRNAs in cardiovascular disease is not fully understood.

In this study, we downloaded microarray data of exosome-derived circRNAs from exoRBase^1^ and microarray data of mRNAs involved in acute myocardial infarction (GSE61144) and identified the differentially expressed genes (DEGs) (26). The differentially expressed circRNAs and mRNAs were predicted using the microRNA target prediction websites (Targetscan, StarBase, miRanda) and the relationship between the two and microRNA. Furthermore, starBase and Circular RNA Interactive were used to identify the corresponding circ-microRNA and micro-mRNA (27, 28). Then, systematic Gene Ontology (GO) and Kyoto Encyclopedia of Genes and Genomes (KEGG) pathway analysis were performed with the DEGs identified following the analysis of the mRNAs (29, 30). STRING was used to study the protein interactions between these DEGs (31), ROC curves were used to verify the diagnostic effect of circRNAs on patients with CHD (32). Finally, we extracted peripheral blood from healthy and CHD individuals, and verified the above-derived RNAs by PCR. In conclusion, our study provides new insights into the potential of circRNAs as diagnostic biomarkers for patients with CHD based on exosomes as delivery vehicles for genes.

## 2. Experimental methods

### 2.1. Microarray data

The expression profiles of exosomal RNA, including circRNA, in blood samples of patients suffering from CHD (6 cases) and healthy individuals (32 cases) were downloaded from the exoRBase (see text footnote 1). mRNA expression profiles in blood samples of patients with acute myocardial infarction were downloaded from the GEO (GSE61144) database, including 7 datasets of patients with acute myocardial infarction and 10 datasets of blood samples from healthy individuals.

### 2.2. Filtering DEGs

Differentially expressed genes (DEGs) screening: “Limma” and “sva” R packages were used to identify the DEGs. RNA conforming to /log2FC/ > 0 and *P* < 0.05 were designated as DEGs, including DEmRNAs and DEcircRNAs, and the corresponding volcano map was generated.

### 2.3. Constructing the ceRNA network

This study was conducted using Target Scan Human 7.2^2^ and miRanda^3^ to predict the microRNA corresponding to the mRNA. ENCORI^4^ was used to predict the corresponding microRNAs of the circRNA. Circular RNA interactive identified corresponding circ-microRNA and micro-mRNA targets. Finally, the prediction data of circRNA with differential expression in the exosomes of patients diagnosed with CHD, mRNA derived from the blood of patients with acute myocardial infarction and their corresponding microRNA were obtained to construct a circRNA-microRNA-mRNA-related ceRNA regulatory network.

### 2.4. Functional enrichment analysis

To evaluate the potential biological functions of the differentially expressed mRNAs corresponding to the circRNA in exosomes, we performed Gene Ontology (GO) annotation and Kyoto Encyclopedia of Genes and Genomes (KEGG) pathway enrichment analysis using clusterProfiler, EnrichPlot, and GGploT2 softwares. GO enrichment analysis primarily included biological process (BP), cell component (CC), and molecular function (MF) groups, and the significance analysis was conducted based on this database. KEGG enrichment analysis was used to analyse the pathway significance of differentially expressed genes based on the KEGG database.

### 2.5. The general information of patients

Thirty-one patients with CHD, who were being treated in the Department of Cardiology of the Second Affiliated Hospital of Harbin Medical University, were selected for the study. Twenty-four arrhythmia or other non-CHD patients of the same gender and age were selected at the same period. The clinical data are shown in Supplementary Table 1. The diagnostic criteria for CHD were set according to the report “Nomenclature and Diagnostic Criteria for Ischemic Heart Disease” formulated by the Joint Task Group on “Clinical Nomenclature Standardization of the International Cardiology Societies and Associations” and the World Health Organization (WHO). The criteria for excluding patients with severe infection and complications and coronary angiographic features were determined by a combination of history, physical examination, serological examination, and angiography with stenosis greater than or equal to 50%. This study was approved by the Ethics Committee of the Second Affiliated Hospital of Harbin Medical University (KY2022-072). All patients participating in the study provided informed consent.

### 2.6. Diagnostic merit of characteristic biomarkers in CHD

To test the efficiency of the prediction of CHD by differential expression of circRNA, we generated receiver operating characteristic (ROC) curves using circRNA expression data from the dataset of 31 CHD patients and 24 healthy individuals. The area under the ROC curve (AUC) was used to evaluate the diagnostic value of the identified hub genes.

### 2.7. qRT-PCR verification

Quantitative reverse transcription polymerase chain reaction (qRT-PCR) was used to detect the variations of gene expression levels in peripheral blood with specific primers. The experiment was performed with biological triplicates. The reaction system was 10 μl. β-actin was used as the internal control. Quantitative analysis of differential expression was assessed by qRT-PCR using SYBR green reaction system on qRT-PCR machine (Bio-Rad, Hercules, CA, USA). The relative expression levels of circRNA were calculated by the 2^–△△Ct^ method. Primer sequences used for qRT-PCR are enlisted in Supplementary Table 2.

### 2.8. Statistical analysis

All statistical analyses related to the bioinformatics study were assessed using the R 3.6.3 statistical software. Graphpad prism 7.00 statistics Mann-Whitney test was used to analyse differences in PCR of circRNA between the CHD group and the healthy control group. Multiple alterations in circRNA expression were assessed by comparing the gene expression levels of patients with CHD and the control group. Screening criteria to examine the differential expression of circRNA was set to *P* < 0.05.

## 3. Results

### 3.1. DEG screening results

**FIGURE 1:**
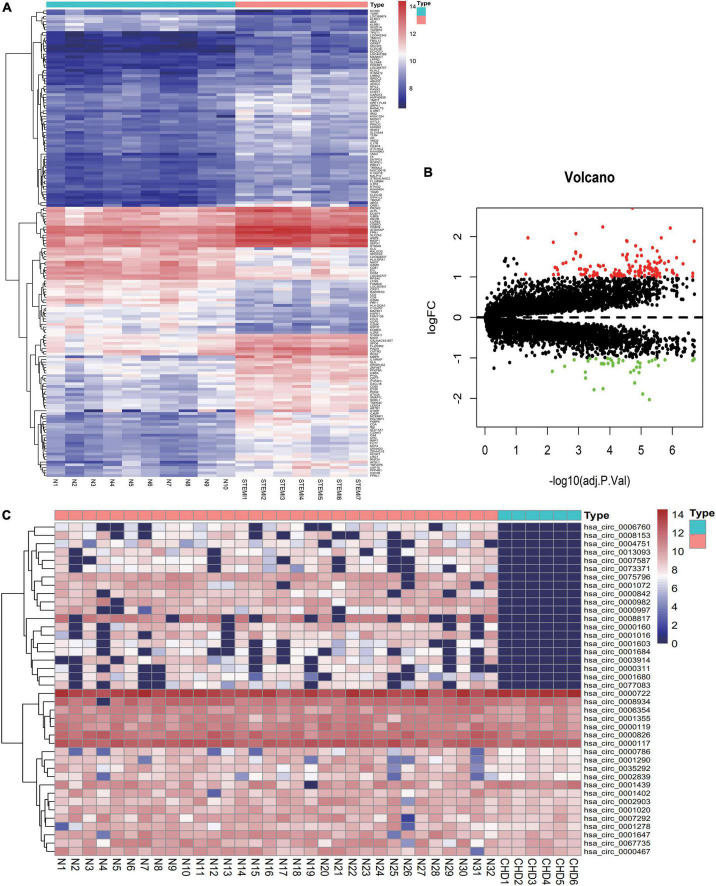
Distribution of differentially expressed genes. **(A)** Heat maps of differentially expressed genes in plasma derived from patients with acute myocardial infarction were divided into acute myocardial infarction (ACS) group (7 cases) and healthy control group (10 cases). Blue to red successively represent gene expression levels from low to high. **(B)** Volcano map of ACS differentially expressed genes. **(C)** Heat maps of differentially expressed circRNA genes in patients with CHD derived from exosomes were divided into CHD group (6 cases) and healthy control group (32 cases). Blue to red represent gene expression from low to high.

Based on the analysis of circRNAs in exosomes from patients with CHD and healthy subjects, 85 differential expressed circRNAs were screened, including 4 up-regulated genes and 81 down-regulated genes. From the analysis of peripheral blood mRNAs in patients with acute myocardial infarction, 173 differentially expressed mRNAs, including 133 up-regulated genes and 40 down-regulated genes, were screened. The DEG heat map and volcano map are shown in Figure 1.

### 3.2. Enrichment analysis of GO terms and KEGG pathways

**FIGURE 2:**
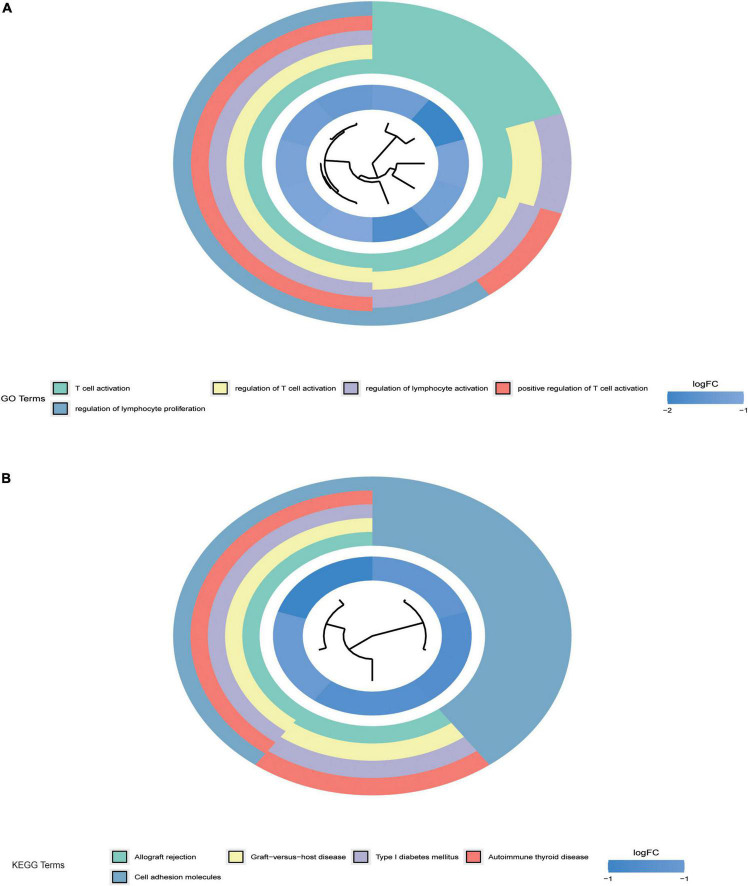
Gene ontology (GO) and kyoto encyclopedia of genes and genomes (KEGG) enrichment analysis. **(A)** Shows the enrichment results of GO pathway analysis of differential genes. **(B)** Shows the enrichment results of KEGG pathway analysis of differential genes. *P* < 0.05 indicated that the enrichment pathway was statistically significant.

To further detect the potential function of differentially expressed mRNAs in acute myocardial infarction in CHD, we performed the GO term and KEGG pathway functional enrichment analyses. The GO term enrichment analysis showed that exosomal mRNAs were mainly enriched in T cell activation, regulation of T cell activation, regulation of lymphocyte activation, positive regulation of T cell activation, and regulation of lymphocyte proliferation (Figure 2). The KEGG pathway enrichment analysis showed that the differentially expressed mRNAs in the regulatory network are mainly enriched in Allograft rejection, Graft-versus-host disease, Type I diabetes mellitus, Epicardial thyroid disease, and Cell adhesion molecules. Studies have shown that CHD is correlated with cell adhesion molecules (32, 33). These results suggest that the differentially expressed circRNAs may play an important role in the occurrence and development of CHD.

### 3.3. STRING analysis of protein interactions

**FIGURE 3:**
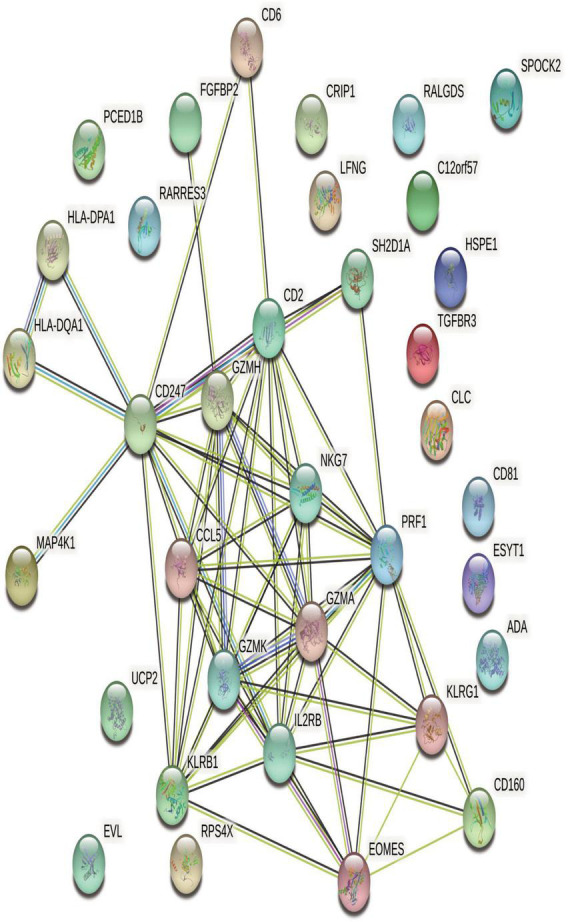
STRING network of protein-protein interactions (PPI) of differentially expressed genes (DEGs) related to AMI. In PPI networks, there are seven different colored lines: 1. Light blue: supported by the evidence from database; 2. Purple: experimentally proved; 3. Red: gene-gene fusion; 4. Yellow-green: supported by the evidence from previous studies; 5. Green: genetic proximity; 6. Blue: co-existing genes; 7. Black: co-expression between genes. Filled nodes indicate the proteins which have their three-dimensional structure known or predicted, while empty nodes designate those whose three-dimensional structures are yet to be resolved.

STRING is an online biological database that offers gene analysis and constructs networks of gene interactions at the protein level (31). In this study, we used STRING (version 11.0) to construct the PPI network of DEGs. To further explore central genes related to CHD and their mechanism of action, 40 genes with down-regulated expression among the 173 differentially expressed genes in the CHD group were identified and uploaded to STRING online database to build a PPI network. A PPI network with 40 nodes and 80 edges was obtained (Figure 3). The nodes represent differentially expressed genes enriched in the STRING database, whereas the edges reflect the interactions between differentially expressed genes. As genes with a high binding degree and high clustering coefficient are important in maintaining the stability of the entire network, we searched for genes with a high binding degree and a clustering coefficient greater than 0.4 using the PPI network. The average node degree was 4.57, average local clustering coefficient was 0.453, and *P*-value of PPI enrichment was <1.0e–16.

### 3.4. circRNA-related ceRNA regulatory network construction

**FIGURE 4:**
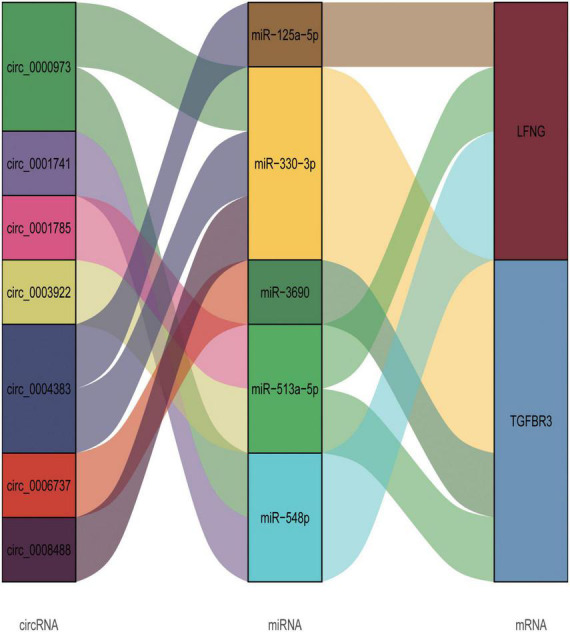
circRNA-microRNA-mRNA related ceRNA regulatory network. In this figure, the first column represents the differentially expressed exosomal circRNAs from patients with CHD, the third column depicts the mRNAs from patients with acute myocardial infarction with differentially expressed mRNAs, and the second column denotes the microRNAs with intersection with circRNA and mRNA as predicted by bioinformatic analysis.

**FIGURE 5:**
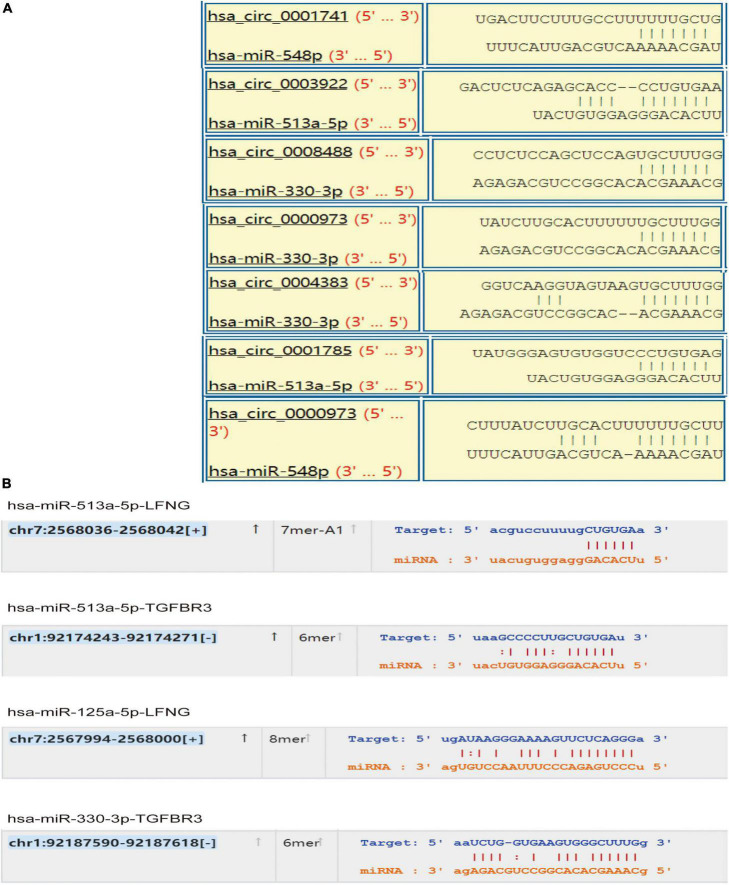
Target prediction of circ-microRNA and micro-mRNA. To further corroborate the accuracy of the target of the ceRNA network, we predict the target location using bioinformatic analysis. **(A)** Binding target of circRNA and microRNA, and **(B)** predicted target binding site of microRNA and mRNA.

Based on the prediction data of circRNAs, mRNAs, and corresponding microRNAs with differential expression in CHD, we constructed a ceRNA network with 7 circRNA nodes, 5 microRNA nodes, and 2 mRNA nodes (Figure 4). To prove the reliability of the ceRNA network, we obtained the corresponding target information of circRNAs, microRNAs, and mRNAs from StarBase, Circular RNA Interactive (Figure 5).

### 3.5. Diagnostic effect of characteristic biomarkers on CHD

**FIGURE 6:**
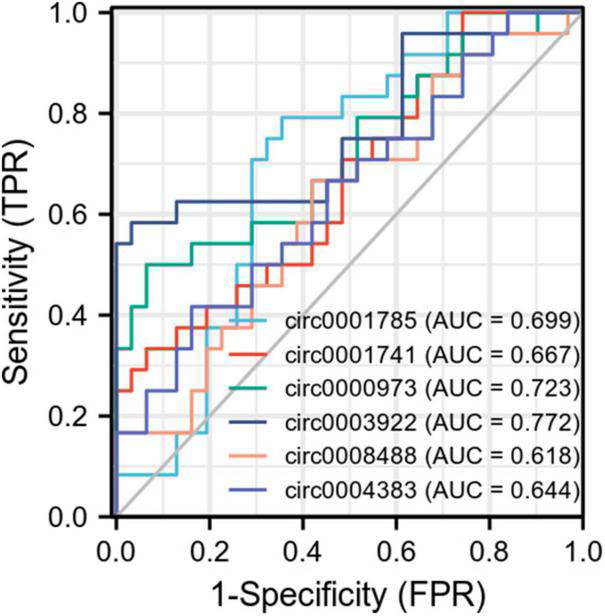
Receiver operator characteristic (ROC) curve of circRNA for the diagnosis of coronary heart disease (CHD). As shown in the figure, each point on the curve corresponds to the FPR and TPR at different thresholds. TPRate refers to the proportion of all samples of true category 1 that are predicted to be in category 1. FPRate refers to the percentage of all samples with true category 0 that are predicted to be in category 1. AUC refers to the random selection of a positive sample and a negative sample from a given category. The classifier predicted that the probability of a positive sample being positive was P1 and that of a negative sample being positive was P2. AUC is the probability that P1 > P2.

Six biomarkers were used to distinguish CHD from the prediction of high diagnostic value in healthy samples (Figure 6). The AUC value of circ0004383 was 0.644 (95% CI = 0.495–0.792), AUC of circ0001741 was 0.667 (95% CI = 0.521–0.812), AUC of circ0000973 was 0.723 (95% CI = 0.583–0.863), ACU of circ0008488 was 0.618 (95% CI = 0.467–0.769), AUC of circ0003922 was 0.772 (95% CI = 0.638–0.905), and AUC of circ0001785 was 0.699 (95% CI = 0.557–0.840). Finally, we produced another joint ROC curve for each circRNA, which showed an area under the curve of 0.784 (Supplementary Figure 1).

### 3.6. PCR validation and analysis results

**FIGURE 7:**
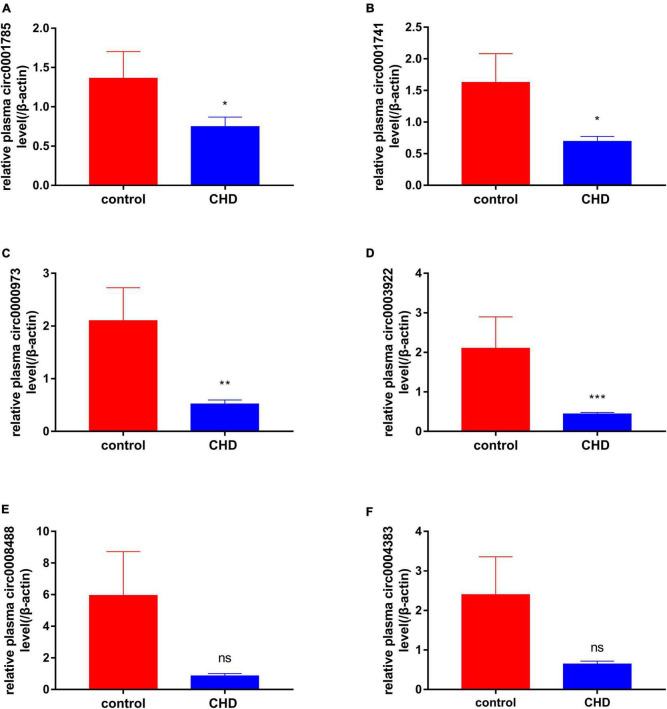
qRT-PCR results of peripheral blood-derived circRNA. The expressions of peripheral blood-derived circRNAs were further confirmed using qRT-PCR. **(A)** Represents the expression of circ0001785 in white blood cells of healthy and coronary patients, **(B)** represents the expression of circ0001741 in white blood cells of healthy and coronary patients, **(C)** represents the expression of circ0000973 in white blood cells of healthy and coronary patients, **(D)** represents the expression of circ0003922 in white blood cells of healthy and coronary patients, **(E)** represents the expression of circ0008488 in white blood cells of healthy and coronary patients, and **(F)** represents the expression of circ0004383 in white blood cells of healthy and coronary patients. Red represents healthy control group, blue represents coronary heart disease (CHD) group, *P* < 0.05 was statistically significant. *P < 0.05, **P < 0.01, and ***P < 0.001.

**FIGURE 8:**
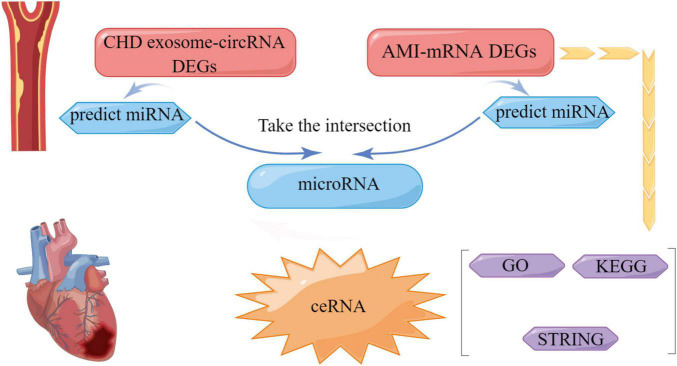
Flow chart of coronary heart disease (CHD).

After screening the differentially expressed circRNAs, we performed PCR verification of circRNA0000973, circRNA0001741, circRNA0001785, circRNA0003922, circRNA0004383, and circRNA0008488. RNA sequence information is shown in Supplementary Table 2. We designed primers for circRNA0006737 for three times, but the results failed to reach the peak value in the qRT-PCR experiment, so we did not conduct further experiments on it. The results showed that the expression of circRNA0001785, circRNA0000973, circRNA0004383, and circRNA0001741 were down-regulated in patients with CHD on peripheral blood. While the expression of circRNA0001785 in CHD was significantly reduced, which was statistically significant. These results validate the results of our bioinformatics analysis (Figure 7). Finally, we built a flow chart for the whole experiment design (Figure 8).

## 4. Discussion

CAD is associated with high morbidity and mortality rates worldwide. In recent years, the number of patients with CHD has been increasing. Thus, there is a need to control the number of patients with CHD and explore its molecular mechanism. Studies have shown that in acute myocardial infarction, myocardial cells secrete exosomes rich in tumor necrosis factor, which leads to myocardial cell damage. On the contrary, cardiac stem-cell-derived exosomes can reduce scar tissue formation (34). Exosome microRNAs play a major role in limiting the development of atherosclerosis (35). However, the role of exosome-derived circRNAs in coronary artery disease is still poorly studied.

With the development of gene chip technology, microarray analysis has been applied in the study of exosomes from patients with CHD. Here, the GEO gene expression dataset was used to detect the differential gene expression between exosomes from patients with CHD and those from healthy subjects, and the ceRNA network of circRNA, mRNA, and the corresponding microRNA was constructed using the bioinformatic prediction website. The GO term and KEGG pathway enrichment analysis were performed on mRNAs differentially expressed in acute myocardial infarction to determine the underlying mechanism of acute myocardial infarction in CHD. Potential biomarker genes were preliminarily verified using the ROC curve. To further confirm the diagnostic function of exosome-derived circRNAs in CHD, basic experiments were performed to verify them. PCR analysis was performed on circRNAs from the healthy and disease groups. Our study contributes to a better diagnosis of CHD and offers potential biomarker for predicting the risk of CHD in acute myocardial infarction.

Finally, through differential expression analysis and functional basis verification, we found that the down-regulation of circRNA0001785, circRNA0000973, circRNA0001741, and circRNA0003922 was statistically significant. Also, we constructed a ceRNA network corresponding to circRNAs related to acute myocardial infarction. The increase in circ0001785 promoted the combination of circ0001785 and miR513a-5p and reduced the combination of miR513a5p and TGFBR3, thus leading to the increased expression of TGFBR3. Similarly, the increased circ0000973 promoted the expression of LFNG by competitively combining with miR-330-3p and miR-548p. The increased circ0001741 that is prone to combine with miR-548p caused the increased expression of LFNG. Also, the increased circ0003922 promoted the expression of TGFBR3 or LFNG by competitively combining with miR-513a-5p. Studies have shown that up-regulated circ0001785 expression in osteosarcoma cells can enhance its carcinogenic effect by up-regulating HoxB2 by sponge miR-1200 (36). Circular RNA hsa_circ_0001785 inhibits the proliferation, migration, and invasion of breast cancer cells *in vitro* and *in vivo* by sponging miR-942 to up-regulate SOCS3 (37). miR-125a-5p can induce the release of gastrin from vascular endothelial cells and thus affect gastrointestinal function (38). miR-513a-5p can mediate TNF-α and LPS-induced apoptosis by down-regulating X inhibitors of apoptosis proteins in endothelial cells (39). TGFBR3 signal can conduct and regulate apoptosis of myocardial cells after infarction. In addition, TGFBR3 signaling is a potential negative regulator that can protect myocardial cell-induced apoptosis (40). Because of the role of these genes in CHD, we confirm that exosome-derived circRNAs can serve as a stable biomarker for diagnosing plaque stability in patients with CHD in future clinical applications.

There are some limitation in this study. First, we did not compare microRNA and mRNA expression in the plasma of patients. This will be further verified in our subsequent experiments. Second, we used bioinformatic methods to infer that exosome-derived circRNA0001785, circRNA0000973, circRNA0001741, and circRNA0003922 can predict the possibility of myocardial infarction in patients with CHD, and these results need to be verified in large-scale studies. Third, according to the current results, the expression of these circRNAs are all at a lower diagnostic level in the ROC curve, but their specificity is higher and their expression is more stable in humans, also its combined ROC curve has a high diagnostic efficiency, so we believe that they are still of great clinical importance.

In conclusion, we found that circRNA0001785, circRNA0000973, circRNA0001741, and circRNA0003922 are potential biomarkers for the diagnosis of CHD, and can affect the stability of plaque by down-regulating these genes. This study provides new insight for studying pathogenesis and prevention strategies of acute myocardial infarction in patients with CHD.

## Data availability statement

Publicly available datasets were analyzed in this study. This data can be found here: https://www.ncbi.nlm.nih.gov/geo/query/acc.cgi?acc=GSE61144.

## Ethics statement

Ethical review and approval was not required for the study on human participants in accordance with the local legislation and institutional requirements.

## Author contributions

XT: conceived and designed the study, conducted the experiments, and analyzed the data. XZ and XD: interpreted the results and prepared the charts. YK and JK: drafted the manuscript and edited it. All authors contributed to the article and approved the submitted version.

## Footnotes

1
http://www.exorbase.org/

2
https://www.targetscan.org/vert_72/

3
cbio.mskcc.org/miRNA2003/miranda.html

4
http://starbase.sysu.edu.cn/
