# Supplementary material for: circRNA, a novel diagnostic biomarker for coronary heart disease
Source: Front Cardiovasc Med. 2023 Feb 1;10:1070616. doi: 10.3389/fcvm.2023.1070616 (PMC9928865; doi:10.3389/fcvm.2023.1070616)
Supplement: Supplementary file 1 [file Data_Sheet_1.PDF]

Supplementary Material

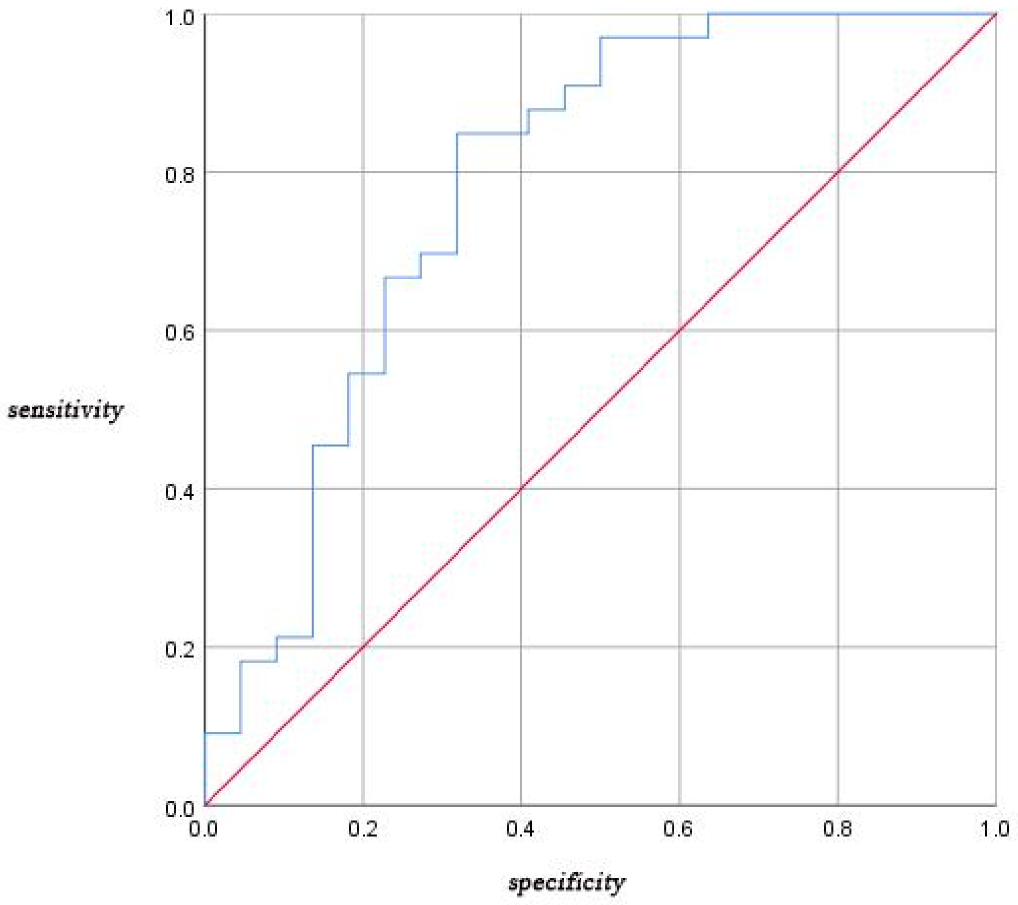

Figure S1. ROC combined with diagnostic probability**.**

Characteristic	CHD(n=31)	control(n=24)
Age, y	62.68±11.27	49.96±15.34
Gender, M/F	21/10	15/9
Total cholesterol (mmol/L)	4.208±0.9917	4.391±1.09
Triglycerides (mmol/L)	1.566±0.6568	1.42±0.6318
LDL (mmol/L)	2.555±0.1622	2.817±0.1952
D-dimer (ng/ml)	139.9±95.87	164.4±252.7
Fibrinogen (g/L)	3.003±0.7533	2.706±0.4012
APTT (sec)	40.36±28.17	34.45±2.637
PT (sec)	10.87±0.6503	12.59±3.381
Platelets ($\times 10^9$/L)	220.9±54.27	209.6±55.64
Leukocytes ($\times 10^9$/L)	8.152±3.101	6.988±2.094
Neutrophils (%)	71.44±2.211	70.99±1.832
Monocytes (%)	2.871±1.274	2.963±1.557
Lymphocytes (%)	23.94±1.866	24.92±1.57
Previous PCI (n)	23	0
Hypertension (n)	17	6
Diabetes mellitus (n)	10	2
Cigarette smoking (n)	7	5
PRO-BNP (pg/ml)	1215±2077	3337±9555
Uric Acid (umol/L)	342.1±98.42	325.2±105.5
glycosylated hemoglobin (%)	7.133±1.829	5.774±0.7723

Table S1. Basic clinical patient information.

Gender, age, diagnosis, hypertension, diabetes, uric acid, white blood cell, neutrophil, glucose and prothrombin time were ordered listed in this table.

Gene name	Species	5’-3’
hsa_circ_0001785	F	ATTTCAGCATCAGGGATTTGGC
	R	GCTCTCTCAGGTTACCATGCTC
hsa_circ_0001741	F	AGCTAATCGGCGCACAGAAA
	R	AGCATGACTCCACATCCTGC
hsa_circ_0003922	F	GCTGAGCCAACAGAGTCCT
	R	GCTGAGCCAACAGAGTCCT
hsa_circ_0008488	F	CACAGTGCTGGGAAGGAATCT
	R	GTGTTCCTGAATTCCTTTAAGTCCA
hsa_circ_0000973	F	TTACAGATATCAGCAGACAGAGGA
	R	GATAAGTTCGAAACCAAATGGCTG
hsa_circ_0004383	F	TCTAGCTCAAGAGACTGGGGTG
	R	AGTTATTTGTGGCTACTGCATCTG
β-actin	F	CTCCATCCTGGCCTCGCTGT
	R	GCTGTCACCTTCACCGTTCC

Table S2. Primer sequence of PCR test.

Primers used in PCR tests are ordered listed in this table.
